# Ocular Signs and Ocular Comorbidities in Monoclonal Gammopathy: Analysis of 80 Subjects

**DOI:** 10.1155/2021/9982875

**Published:** 2021-06-18

**Authors:** Kitti Kormányos, Klaudia Kovács, Orsolya Németh, Gábor Tóth, Gábor László Sándor, Anita Csorba, Cecília Nóra Czakó, Achim Langenbucher, Zoltán Zsolt Nagy, Gergely Varga, László Gopcsa, Gábor Mikala, Nóra Szentmáry

**Affiliations:** ^1^Semmelweis University, Department of Ophthalmology, Budapest, Hungary; ^2^Medical Centre, Hungarian Defence Forces, Department of Ophthalmology, Budapest, Hungary; ^3^Markusovszky University Teaching Hospital, Department of Ophthalmology, Szombathely, Hungary; ^4^Experimental Ophthalmology, Saarland University, Homburg, Saarland, Germany; ^5^3^rd^ Department of Internal Medicine and Haematology, Semmelweis University, Budapest, Hungary; ^6^South-Pest Central Hospital–National Institute for Hematology and Infectious Diseases, Department of Hematology and Stem Cell-Transplantation, Budapest, Hungary; ^7^Dr. Rolf M. Schwiete Center for Limbal Stem Cell and Congenital Aniridia Research, Saarland University, Homburg, Saarland, Germany

## Abstract

**Purpose:**

To examine the ocular signs of monoclonal gammopathy and to evaluate ocular comorbidities in subjects with monoclonal gammopathy. *Patients and Methods*. We analyzed patients from two large referral hematology centers in Budapest, diagnosed and/or treated with monoclonal gammopathy between 1997 and 2020. As a control group, randomly selected individuals of the same age group, without hematological disease, have been included. There were 160 eyes of 80 patients (38.75% males; age 67.61 ± 10.48 (range: 38–85) years) with monoclonal gammopathy and 86 eyes of 43 control subjects (32.56% males; age 62.44 ± 11.89 (range 37–86) years). The hematological diagnosis was MGUS in 9 (11.25%), multiple myeloma in 61 (76.25%), smoldering myeloma in 6 (7.50%), and amyloidosis or Waldenström macroglobulinemia in 2 cases (2.50%–2.50%). Before detailed ophthalmic examination with fundoscopy, 42 subjects with gammopathy (52.50%) and all controls filled the Ocular Surface Disease Index (OSDI) questionnaire.

**Results:**

The OSDI score and best-corrected visual acuity (BCVA) were significantly worse in subjects with monoclonal gammopathy than in controls (*p*=0.02; *p*=0.0005). Among gammopathy subjects, we observed potential corneal immunoglobulin deposition in 6 eyes of 4 (3.75%) patients. Ocular surface disease (*p*=0.0001), posterior cortical cataract (*p*=0.01), and cataract (*p*=0.0001) were significantly more common among gammopathy subjects than in controls (*χ*^2^ test).

**Conclusions:**

Ocular surface disease and cataract are more common, and BCVA is worse in patients with monoclonal gammopathy than in age-matched controls. Therefore, and due to the potential ocular signs and comorbidities of monoclonal gammopathy, we suggest a regular, yearly ophthalmic checkup of these patients to improve their quality of life.

## 1. Introduction

The spectrum of monoclonal gammopathies spans clonal plasma cell diseases from monoclonal gammopathy of undetermined significance (MGUS), solitary plasmacytoma, Waldenström macroglobulinemia, and asymptomatic or symptomatic multiple myeloma to plasma cell leukemia [1–3]. MGUS is considered a premalignant state that has three different types with IgM MGUS, non-IgM MGUS (IgA- and IgG-MGUS), and light chain MGUS. All forms of MGUS can cause amyloidosis, a special sort of light chain deposition disease, or non-Hodgkin lymphoma, which are important differential diagnostic entities [4].

Diagnostic criteria for MGUS according to the 2015 recommendation of the International Myeloma Working Group are bone marrow plasma cell content less than 10%, less than 3 g/dL of monoclonal protein level (M-protein) in the serum, and no indication of organ disruption, that is characteristic for malignant B-cell disease (no hypercalcemia, renal failure, anemia, or bone changes) [5–7]. However, with IgG-type M-protein of less than 1.5 g/dL, bone marrow biopsy is often defered if the patient is asymptomatic. The prevalence of MGUS increases with age from 1.7% in individuals with 50–59 years of age to 6.6% in individuals with >80 years of age (Kyle et al. 2006) [8].

In monoclonal gammopathy, monoclonal protein deposition has been described in different organs in the literature [9]. In multiple myeloma, kidneys are involved up to 40% [10], and unexplained polyneuropathy is present in 5–10% [11–13]. There may also be insulin autoimmune syndrome [14], infiltrative or restrictive cardiomyopathy [15], gastrointestinal system involvement [16], and infiltrative or “paraneoplastic”-like skin disease [17].

As ocular signs of gammopathy, corneal deposits, conjunctival deposits, acute/chronic uveitis [18, 19], maculopathy, foveolar drusen [20–22], Doyne retinal dystrophy [23], central retinal artery or vein occlusion [24], myositis, and proptosis [25] have been described.

Corneal deposits associated with monoclonal gammopathy (or paraproteinaemia) have been first described in the 1900s as chameleon-like changes [26, 27]. These are mostly bilateral, grey-white, yellowish, grey-brown, polychromatic, or crystal-like changes in any layer of the cornea. These may be either diffuse or focal, central or peripheral deposits [5]. As these corneal deposits in MGUS may appear in countless forms, they have been termed “chameleon-like” corneal depositions by Lisch and his coworkers. They created a uniform and internationally accepted nomenclature in 2012, which distinguished 5 different types of immunotactoid keratopathy (ITK): crystalline-like ITK, lattice-like ITK, peripheral granular-like ITK, peripheral band-like ITK, and peripheral patch-like ITK. Later in 2016, they expanded the classification system to 11 distinct forms of MGUS-induced paraproteinemic keratopathy [27].

However, data on the occurrence of ocular signs of monoclonal gammopathy and on ocular comorbidities are not yet described. We aimed to determine the ocular signs of monoclonal gammopathy and the ocular comorbidities in subjects with monoclonal gammopathy.

## 2. Patients and Methods

In our perspective, it is a cross-sectional study in which we analyzed patients of the Department of Hematology and Stem Cell-Transplantation of the South-Pest Center Hospital, National Institute for Hematology and Infectious Disease, Budapest, Hungary, and the 3rd Department of Internal Medicine, Semmelweis University, Budapest, Hungary, diagnosed and treated with monoclonal gammopathy between 1997 and 2020. As a control group, randomly selected individuals of the same age group, without hematological disease, have been included. The local ethics committee gave permission to our study (OGYÉI/50115/2018). Participation in this study has been voluntary, and written informed consent was obtained from all participants.

In our study, we analyzed altogether 246 eyes of 123 patients (age 66.2 ± 11.11 years). There were 160 eyes of 80 patients (38.75% males; age 67.61 ± 10.48 (range: 38–85) years) with monoclonal gammopathy. Eighty-six eyes of 43 subjects (32.56% males; age 62.44 ± 11.89 (range: 37–86) years) have been analyzed as controls. The age of the patients in the gammopathy and control groups did not differ significantly (*p*=0.17) (Table 1).

In patients with established hematological diagnosis, the time of the hematological diagnosis was in one case (1.25%) within 1 year, in 36 (45.00%) cases within 5 years, in 29 (36.25%) cases within 5–10 years, and in 14 (17.50%) cases more than 10 years ago. The hematological diagnosis was MGUS in 9 (11.25%), multiple myeloma in 61 (76.25%), smoldering myeloma in 6 (7.50%), and amyloidosis or Waldenström macroglobulinemia in 2 cases (2.50%).

With respect to immunoglobulin heavy chains, there was an increased IgG level in 52 individuals (65.00%), an increased IgA level in 20 (25.00%), an increased IgM level in 4 (5.00%), and an increased IgD level in 1 case (1.25%). Considering light chains, kappa chain in 49 subjects (61.25%), and lambda chain in 31 patients (38.75%) have been verified, and in 3 cases (3.75%), heavy chain production was not detectable.

With respect to organ dysfunction in gammopathy patients, osteolytic lesions have been previously described in 39 subjects (48.75%), renal involvement in 19 patients (23.75%), polyneuropathy in 6 cases (7.50%), spinal cord involvement in 3 cases (3.75%), and liver involvement and thrombosis of the upper limb in 2 cases (2.5%), respectively. In single cases, infiltration of the nervus medianus and skin lesions (1.25%) were identified. There was no renal involvement in 4 (5.00%) and no other organ involvement in 8 (10.00%) subjects in the hematological disease history. 36 subjects (45.00%) had previous autologous stem cell transplantation, and 65 subjects (81.25%) received chemotherapy, according to their hematological disease history.

In the gammopathy group, there was hypertension in 59 (73.75%), type 2 diabetes mellitus in 15 (18.75%), cardiac arrhythmia in 10 (12.50%), gastro-oesophageal reflux in 9 (11.25%), previous myocardial infarction in 4 (5.00%), deep vein thrombosis in 4 (5.00%), stroke in 3 (3.75%), benign prostate hyperplasia in 3 (3.75%), prostate cancer in 3 (3.75%), cervix cancer in 3 (3.75%), hyperthyroidism in 3 (3.75%), asthma bronchiale in 2 (2.50%), breast cancer in 2 (2.50%), Raynaud's syndrome in 2 (2.50%), rheumatoid arthritis in 2 (2.50%), hypothyroidism in 2 (2.50%), endometriosis in 1 (1.25%), pulmonary embolism in 1 (1.25%), systemic lupus erythematosus in 1 (1.25%), colon cancer in 1 (1.25%), endometrial cancer in 1 (1.25%), and squamous cell skin cancer in 1 (1.25%) subject.

In the control group, there was hypertension in 16 (37.21%), type 2 diabetes in 6 (13.95%), atrial fibrillation in 2 (4.65%), gastro-oesophageal reflux in 1 (2.32%), prostate cancer in 1 (2.32%), and colon cancer in 1 (2.32%) subject in history, respectively.

Before the ophthalmic examination, 42 subjects with gammopathy (52.50%) and all control subjects filled the Ocular Surface Disease Index (OSDI) questionnaire (score ranges were designated as normal (0–12), mild (13–22), moderate (23–32), or severe (33–100) ocular surface disease), and for all patients, ophthalmic medical history has been taken. Ophthalmic examination included refractometry, visual acuity test (best-corrected visual acuity), Goldmann applanation tonometry, and slit-lamp examination following dilation of the pupil. In the case of retinal disease, optical coherence tomography (AngioVue OCTA, RTVue XR Avanti, OptoVue, Fremont CA, USA) has also been performed.

For statistical analysis of the data, the Mann–Whitney *U*-test and *χ*^2^ test have been used.

## 3. Results

Ophthalmic history and results of the ophthalmic examination in patients with monoclonal gammopathy and in control subjects are summarized in Tables 2 and 3.

In the ophthalmic history of subjects with monoclonal gammopathy, there was no history of ocular disease in 66 (41.25%), and there was dry eye disease in 64 (40.00%), cataract in 27 (16.88%), previous cataract surgery in 20 (12.50%), glaucoma in 12 (7.50%), posterior cortical cataract in 4 (2.50%), and previous penetrating keratoplasty in 2 eyes (1.25%). In the subgroup of the 8 subjects with MGUS (16 eyes, without previous systemic corticosteroid treatment), there was a cataract in 14 (77.77%) and posterior cortical cataract in 4 (22.23%) eyes. None of them had previous cataract surgery.

In the ophthalmic history of controls, there was no history of ocular disease in 22 (25.58%), and there was dry eye disease in 17 (19.77%), previous cataract surgery in 12 (13.95%), cataract in 12 (13.95%), glaucoma in 4 (4.65%) and posterior cortical cataract in 1 (1.16%) eyes.

In ophthalmic history, the proportion of subjects with dry eye disease was significantly higher in monoclonal gammopathy subjects as in controls (*p*=0.002).

Using the OSDI questionnaire, among patients with hematological diagnosis, there were 14 (33.33%) subjects with normal ocular surface, 11 (26.19%) had mild, 6 (14.29%) had moderate, and 11 (26.19%) had severe ocular surface disease. Among the control subjects, there were 27 subjects (62.79%) with normal ocular surface, 7 subjects (16.28%) with mild, 7 (16.28%) with moderate, and 2 (4.65%) with severe ocular surface disease. OSDI score was significantly worse in subjects with monoclonal gammopathy than in controls (*p*=0.02).

In patients with hematological diagnosis, best-corrected visual acuity (BCVA) was 0.82 ± 0.26 (logMAR 0.1 ± 0.26), and in controls, it was 0.94 ± 0.16 (logMAR 0.1 ± 0.16). BCVA was significantly worse in subjects with gammopathy than in controls at the examination time-point (*p*=0.0005).

Among patients with gammopathy, we found 89 (55.63%) eyes of 53 patients with 1.0 (0.0 logMAR) and 66 eyes (41.25%) of 42 patients between 0.2 and 0.9 (0.1–0.7 logMAR) BCVA. Five (3.13%) eyes of 5 patients were not able to read the chart. Between controls, in the majority of the subjects, 68 (79.07%) eyes had BCVA 1.0 (0.0 logMAR), 12 (13.95%) eyes of 10 patients had BCVA 0.8-0.9 (0.1 logMAR), 5 (5.81%) eyes of 5 patients had BCVA between 0.2 and 0.7 (0.2–0.5 logMAR), and 1 (1.16%) eye of 1 patient was not able to read the chart.

Among ophthalmological findings of gammopathy subjects, there was ocular surface disease in 56 (66.67%), cataract in 86 (53.75%), Meibomian gland dysfunction in 30 (18.75%), no ophthalmic disease in 22 (13.75%), posterior cortical cataract in 21 (13.13%), previous cataract surgery in 20 (12.50%), macular or retinal drusen in 18 (11.25%), chronic blepharitis in 16 (10.00%), glaucoma in 12 (7.50%), age-related macular degeneration in 12 (7.50%), epiretinal membrane in 10 (6.25%), Fuchs dystrophy in 8 (5.00%), peripheral retinal degeneration in 7 (4.38%), corneal immunoglobulin deposition in 6 (3.75%), diabetic retinopathy in 4 (2.50%), amblyopia in 3 (1.88%), macular hole in 1 (0.63%), central retinal artery occlusion in 1 (0.63%), branch retinal vein occlusion in 1 (0.63%), choroidal nevus in 1 (0.63%), and retinal scar in 1 (0.63%) eye.

**Figure 1:**
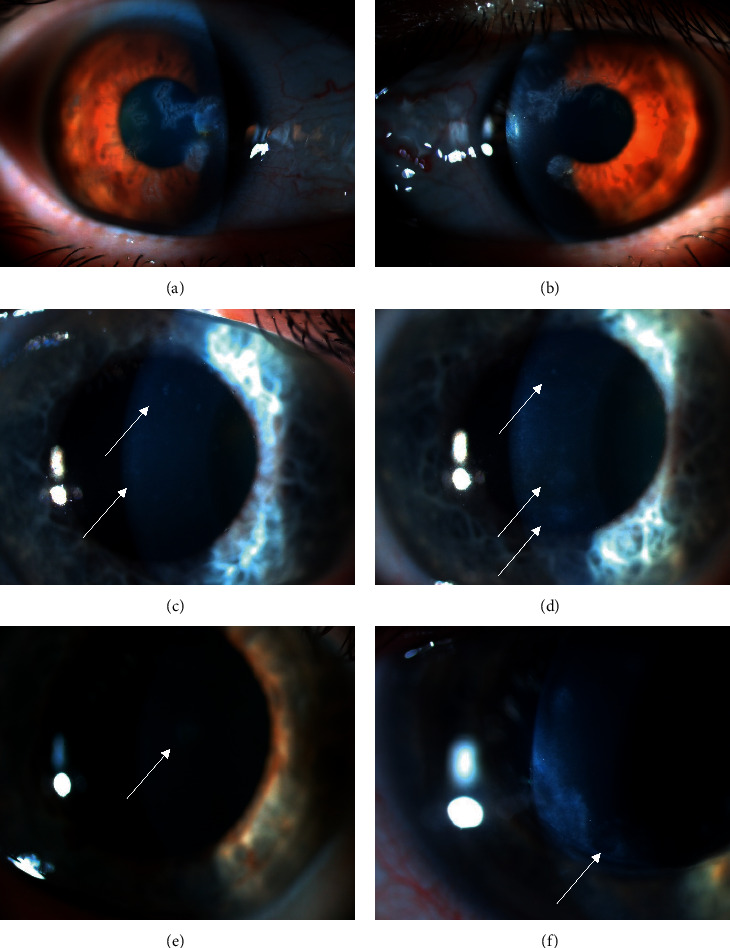
Corneal opacities in six eyes of four patients with monoclonal gammopathy. Sharp-edged, branching predescemetal opacities in both eyes of one patient with monoclonal gammopathy of ocular significance (MGOS) (a, b), sharpe-edged, round or puntual, fine subepithelial opacities in both eyes of one subject with cornea guttata and multiple myeloma (c, d), round stromal opacity in one eye of one subject with multiple myeloma (e), sand-like stromal deposits along the removed penetrating keratoplasty (PKP) running-suture line in one eye of one subject with multiple myeloma (f).

Among gammopathy subjects, we observed potential corneal immunoglobulin deposition in 6 eyes of 4 (7.50%) patients (Figure 1). One of these patients underwent penetrating keratoplasty (PKP) prior to enrollment (Figure 1(f)). These corneal deposits have been observed in both eyes in 2 patients (Figures 1(a)–1(d)) and in 1 eye in 2 patients (Figures 1(e) and 1(f)). The diagnosis was monoclonal gammopathy with ocular significance (MGOS) in 1 (Figures 1(a) and 1(b)) and multiple myeloma in 3 (Figures 1(c)–1(f)) of these subjects.

In gammopathy subjects, in the group of corneal scars and degenerations, there was arcus senilis in 8 (5.00%), crocodile shagreen in 6 (3.75%), iron line and corneal scar due to previous corneal foreign body removal in 5 (3.13%), Salzmann nodular degeneration in 1 (0.63%) and stromal scar and calcification due to previous stromal herpes keratitis in 1 (0.63%) eye.

Between ophthalmological findings of control subjects, there was ocular surface disease in 32 (37.21%), cataract in 17 (19.77%), macular or retinal drusen in 16 (18.60%), chronic blepharitis in 16 (18.60%), no ophthalmic disease in 14 (16.28%), previous cataract surgery in 12 (13.95%), Meibomian gland dysfunction in 10 (11.63%), glaucoma in 4 (4.65%), diabetic retinopathy in 4 (4.65%), peripheral retinal degeneration in 4 (4.65%), posterior cortical cataract in 3 (3.49%), Fuchs dystrophy in 2 (2.33%), epiretinal membrane in 2 (2.33%), and amblyopia in 1 (1.16%) eye.

In control subjects, in the group of corneal scars and degeneration, there was arcus senilis in 2 (2.33%), crocodile shagreen in 2 (2.33%), and iron line and corneal scar due to previous corneal foreign body removal in 1 (1.16%) eye. The proportion of subjects with corneal scars and degenerations in the gammopathy group did not differ from controls (*p*=0.07).

## 4. Discussion

To the best of our knowledge, this is the first study to analyze ocular signs and ocular comorbidities of monoclonal gammopathy. In Hungary, approximately 350–400 new patients are diagnosed and registered with multiple myeloma yearly and 120–150 autologous bone marrow transplantations are performed due to multiple myeloma [28]. The incidence of MGUS is unknown. Most interestingly, ocular surface disease and cataract are more common, and BCVA and OSDI scores are worse (BCVA lower, OSDI scores higher) in patients with gammopathy than in age-matched controls.

In our analyzed cohort with gammopathy with a mean age of 67.61 years and control subjects with a mean age of 62.44 years, the prevalence of ocular surface disease using the OSDI questionnaire was 66.67% and 37.21%, respectively. In the literature, the prevalence of dry eye disease in subjects older than 50 years was described to be 5–34% [29, 30], which confirms our results observed in the control subjects. Nevertheless, in patients with monoclonal gammopathy, the OSDI score and prevalence of ocular surface disease was significantly higher than expected. This could be related to the monoclonal gammopathy itself, or to the previous systemic corticosteroid treatment or chemotherapy which the patients underwent (for malignant plasma cell disorder). Dry eye disease has been previously described as a side effect of these systemic treatment forms [31].

Although the percentage of patients with previous cataract surgery did not differ significantly between both groups, the proportion of subjects with unoperated posterior cortical cataract or cataract was significantly higher in subjects with gammopathy (13.13% vs. 3.49% and 53.75% vs. 19.76%) than in controls (*p*=0.0001 and *p*=0.01). Similarly to dry eye disease, cataract formation has also been associated with systemic corticosteroid treatment (subjects with plasma cell malignancy exhibiting monoclonal gammopathy often receive systemic steroids over months, mostly due to induction therapy before autologous stem cell transplantation) and multiagent chemotherapy, previously [32], but could also be associated with the monoclonal gammopathy itself and the changes in protein metabolism. Chen et al. described cataract prevalence in 6725 subjects older than 50 years 23.1% [33]. In our study population with monoclonal gammopathy, cataract prevalence was more than two times higher than in our control subjects and nearly two times higher than in the study of Chen et al. In addition, cataract prevalence was also similar in the corticosteroid naive MGUS patient group.

Prevalence of chronic blepharitis was described to be 8.1% in subjects older than 40 years by Rim et al. [34]. Similarly, there was chronic blepharitis in 10.0% of gammopathy subjects and its percentage did not differ from our control subjects, or historic data.

The prevalence of Meibomian gland dysfunction was described to be 36% in subjects with 50–59 years of age [35]. Interestingly, Meibomian gland dysfunction was rather uncommon with 11.63% in our control and with 18.75% in our gammopathy groups, without statistically significant difference.

Epidemiological data show that patients with myeloproliferative neoplasms suffer from an accelerated accumulation of subretinal drusen, and this phenomenon is associated with an increased risk of neovascular age-related macular degeneration (AMD) [36]. Immunoglobulin deposition has also been associated with maculopathy and the appearance of foveolar drusen [20]. Although there was AMD in 7.5% of our gammopathy subjects and none in our control subjects, the percentage of macular or retinal drusen did not differ between both groups with 11.25% vs. 18.6%. This needs further analysis.

There are numerous studies in the literature describing corneal deposition in monoclonal gammopathy. Garibaldi et al. [26] in 2004 presented a case report and literature review, summarizing 38 cases with corneal deposition.

In our subjects with monoclonal gammopathy, the suggested immunoglobulin deposition was present in only 3.75%, a relatively low percentage of 160 eyes; however, the ophthalmologists have an essential role in detecting paraproteinemic keratopathy as an ophthalmic sign of the hematological disease, which should never be forgotten.

In our study, 12 eyes of 6 patients (7.50%) with monoclonal gammopathy had glaucoma, 10 eyes (6.25%) had open-angle glaucoma (OAG), and 2 eyes (1.25%) had angle-closure glaucoma (ACG). Bertaud et al. [37] described the prevalence of OAG 3.05% in subjects between 40 and 80 years of age. Wright et al. [38] described the prevalence of ACG 0.02% in subjects with 40–49 years of age, and ACG prevalence increases to 0.95% in subjects older than 70 years. Between our controls, there were 4 eyes (4.65%) with glaucoma. The proportion of glaucoma subjects was slightly higher in monoclonal gammopathy subjects, nevertheless, without a statistically significant difference, compared to our control population. Nevertheless, compared to literature data, both OAG and ACG prevalence was higher in our monoclonal gammopathy subjects as in the general population. Therefore, during an ophthalmic checkup, glaucoma screening should always be performed in monoclonal gammopathy subjects.

Although the percentage of epiretinal membrane was 6.25% among gammopathy subjects, this did not differ significantly from those in controls (2.33%).

In plasma cell disorders, venous thromboembolism is a frequent complication due to hyperviscosity of the blood [24, 39]. This is well displayed in ophthalmic findings in our patients, as we found 1 subject with previous central retinal artery occlusion and 1 with branch retinal vein occlusion. Both entities warrant regular ophthalmic checkups and ophthalmic treatment.

In addition to immunoglobulin deposition in the cornea and conjunctiva, other ophthalmic abnormalities have been reported in monoclonal gammopathy [18]. Some publications report the simultaneous appearance of monoclonal gammopathy and acute or chronic uveitis [19]. Moreover accumulation of monoclonal immunoglobulin crystals (kappa light chain type) in orbital fat and extraocular muscles, causing invasive masses (crystal storage histiocytosis) has been reported. Palpebral ecchymoses can occur due to vascular fragility secondary to amyloid. Munteanu has suggested a connection between Doyne's retinal dystrophy, benign monoclonal gammopathy, and the presence of corneal deposits [23]. Nevertheless, none of these entities were verified in our subjects, referring to the heterogeneity of diseases with monoclonal gammopathy, also concerning ocular signs and ocular comorbidities.

In summary, ocular surface disease and cataract are more common, and BCVA is worse in patients with monoclonal gammopathy than in age-matched controls. Therefore, and due to the potential ocular signs and comorbidities of monoclonal gammopathy, we suggest a regular, yearly ophthalmic checkup of these patients to improve their quality of life.

## Figures and Tables

**Table 1:** Age, gender, Ocular Surface Disease Index (OSDI) score, best-corrected visual acuity (BCVA) in control subjects and in patients with monoclonal gammopathy (MGUS).

—	Age (years) (mean ± SD (minimum-maximum))	Males (*n* (%))	OSDI (mean ± SD (minimum-maximum))	BCVA (mean ± SD (minimum-maximum))
Control	62.44 ± 11.89 (37–86)	14 (32.56%)	12.66 ± 11.00 (0–50)	0.94 ± 0.16 (0.06–1)
MGUS	67.61 ± 10.48 (38–85)	31 (38.75%)	21.51 ± 18.03 (0–65.9)	0.82 ± 0.26 (0.01–1)
*P* value	0.17	0.67	**0.02**	**0.0005**

*P* values refer to results of Mann–Whitney *U*-test (age, OSDI, and BCVA) and of *χ*^2^ test with Yates correction (males) (comparison between both groups). Significant values are in bold.

**Table 2:** Ophthalmic diagnosis in ophthalmic history of control subjects and in patients with monoclonal gammopathy (MG).

Ophthalmic diagnosis	Control (*n* = 86)	MG (*n* = 160)	*P* values
Dry eye disease	17 (19.77%)	64 (40.00%)	**0.002**
Penetrating keratoplasty	0	2 (1.25%)	—
Glaucoma	4 (4.65%)	12 (7.50%)	0.55
Previous cataract surgery	12 (13.95%)	20 (12.50%)	0.90
Cataract	12 (13.95%)	27 (16.88%)	0.54
Posterior cortical cataract	1 (1.16%)	4 (2.50%)	0.47
Without previous ophthalmic diagnosis	22 (25.58%)	66 (41.25%)	**0.01**
Total	**86 (100%)**	**160 (100%)**	—

*P* values refer to results of *χ*^2^ test with Yates correction (comparison between both groups). Significant values are in bold. With “0” value, *χ*^2^ test could not be calculated.

**Table 3:** Ophthalmological findings in control subjects (86 eyes) and in patients with monoclonal gammopathy (MG) (160 eyes, except for ocular surface disease, as OSDI questionnaire has only been filled through 42 subjects).

Ophthalmic diagnosis	Control (*n* = 86)	MG (*n* = 160)	*P* values
Ocular surface disease (OSDI)	32 (37.21%) (*n* = 86)	56 (66.67%) (*n* = 84)	**0.0001**
Meibomian gland dysfunction	10 (11.63%)	30 (18.75%)	0.20
Chronic blepharitis	16 (18.60%)	16 (10.00%)	0.08
Corneal scars and degenerations	5 (5.81%)	21 (13.13%)	0.07
Corneal immunglobulin deposition	0	6 (3.75%)	—
Fuchs dystrophy	2 (2.33%)	8 (5.00%)	0.50
Glaucoma	4 (4.65%)	12 (7.50%)	0.55
Previous cataract surgery	12 (13.95%)	20 (12.50%)	0.90
Cataract	17 (19.76%)	86 (53.75%)	**0.0001**
Posterior cortical cataract	3 (3.49%)	21 (13.13%)	**0.01**
Epiretinal membrane	2 (2.33%)	10 (6.25%)	0.29
Age-related macular degeneration	0	12 (7.50%)	—
Macular or retinal drusen	16 (18.60%)	18 (11.25%)	0.16
Macular hole	0	1 (0.63%)	—
Diabetic retinopathy	4 (4.65%)	4 (2.50%)	0.59
Peripheral retinal degeneration	4 (4.65%)	7 (4.38%)	0.82
Central retinal artery occlusion	0	1 (0.63%)	—
Branch retinal vein occlusion	0	1 (0.63%)	—
Choroideal naevus	0	1 (0.63%)	—
Retinal scar after chorioretinitis	0	1 (0.63%)	—
Ambyopia	1 (1.16%)	3 (1.88%)	0.93
Without ophthalmic disease	14 (16.28%)	22 (13.75%)	0.72
Total	**86 (100%)**	**160 (100%)**	—

*P* values refer to results of *χ*^2^ test with Yates correction (comparison between both groups). Significant values are in bold. With “0” value, *χ*^2^ test could not be calculated. OSDI: Ocular Surface Disease Index.

## Data Availability

The data used to support the findings of this study are available from the corresponding author upon request.
